# Estrogen receptor-beta is a potential target for triple negative breast cancer treatment

**DOI:** 10.18632/oncotarget.26089

**Published:** 2018-09-21

**Authors:** David Austin, Nalo Hamilton, Yahya Elshimali, Richard Pietras, Yanyuan Wu, Jaydutt Vadgama

**Affiliations:** ^1^ Department of Medicine, Division of Cancer Research and Training, Charles Drew University School of Medicine and Science, Los Angeles, CA 90059, USA; ^2^ UCLA School of Nursing, University of California at Los Angeles, Los Angeles, CA 90095, USA; ^3^ UCLA Johnson Comprehensive Cancer Center, University of California at Los Angeles, Los Angeles, CA 90095, USA; ^4^ UCLA David Geffen School of Medicine, Department of Medicine, Division of Hematology-Oncology, University of California at Los Angeles, Los Angeles, CA 90095, USA

**Keywords:** estrogen receptor-beta, triple negative breast cancer, insulin-like growth factor 2, DPN, estrogen receptor-beta signaling

## Abstract

Triple Negative breast cancer (TNBC) is a subtype of breast cancer that lacks the expression of estrogen receptor (ER), progesterone receptor, and human epidermal growth factor receptor 2. TNBC accounts for 15-20% of all breast cancer cases but accounts for over 50% of mortality. We propose that Estrogen receptor-beta (ERβ) and IGF2 play a significant role in the pathogenesis of TNBCs, and could be important targets for future therapy.

Tissue microarrays (TMAs) from over 250 TNBC patients' were analyzed for ERβ and IGF2 expression by immunohistochemistry. Expression was correlated with clinical outcomes. In addition, TNBC cell lines Caucasians (CA): MB-231/BT549 and African Americans (AAs): MB-468/HCC70/HCC1806 were used to investigate the effect of hormonal and growth factor regulation on cell proliferation.

TMAs from AAs had higher expression of ERβ and IGF2 expression when compared to CA. ERβ and IGF2 were found to be upregulated in our TNBC cell lines when compared to other cell types. TNBC cells treated with ERβ agonist displayed significant increase in cell proliferation and migration when compared to controls. AA tissue samples from TNBC patients had higher expression of ERβ. African-American breast cancer TNBC tissue samples from TNBC patients have higher expression of ERβ. In addition, TNBC cell lines were also found to express high levels of ERβ. IGF2 increased transcription of ERβ in TNBC cells. Understanding the mechanisms of IGF2/ERβ axis in TNBC tumors could provide an opportunity to target this aggressive subtype of breast cancer.

## INTRODUCTION

Triple negative breast cancer (TNBC) is a subtype of breast cancer (BC) and is defined by the lack of expression of three receptors: the estrogen receptor (ER), progesterone receptor (PR), and the human epidermal growth factor receptor 2 (HER2) [1]. TNBC accounts for roughly 15% of all breast cancer cases but represents over 50% of mortality seen in breast cancer [2–4]. Therefore, current endocrine and HER2-targeted therapies are not viable for TNBCs, and the only treatment option available is chemotherapy [2, 4–6]. Although, TNBC patients tend to have higher clinical response rates to chemotherapy, they also have higher rates of distant recurrence and a worse overall prognosis than women with other breast cancer subtypes [7, 8]. TNBC is associated with health disparity because it is more common in premenopausal women of color (African-American women (AA) and Hispanic women (HS)) than Caucasian American women (CA) [3, 9]. Population based studies show that AA women with TNBC have a higher incidence, disease stage, and metastasis than CA [10, 11]. Therefore finding a new molecular target and/or treatment is the upmost importance within this patient population and in women with breast cancer in general.

Estrogens promote progression of ER-alpha (ERα) positive cancers, effected by the binding of estradiol to ERα [12]. ERα is predominantly localized in the nucleus and positive staining by immunohistochemical (IHC) detection, helps plan patient management [13]. Recent reports have shown a second ER, ERβ, is expressed in TNBC [14, 15]. ERα and ERβ are encoded by two different genes but share 96% homology in the DNA-binding domain and 60% homology in the ligand binding domain of ERα. ERβ's role in BC progression remains to be elucidated, however, some studies have shown ERβ positivity is a biomarker related to a more aggressive clinical outcome [15] and correlates with Ki-67, a proliferation marker [14–16]. Early studies suggest ERβ levels are higher in BCs in AA as compared to CA, and may play a role in TNBC progression [17–20]. Several ERβ isoforms occur in BC, including ERβ1, ERβ2, ERβ4, and ERβ5. Only ERβ1 retains an intact ligand binding domain which makes it a preferred clinical target [21–23]. ERβ occurs in the nucleus and in the cytoplasm and can be activated by both genomic or indirectly by nongenomic pathways [21, 24–26]. Recent reports indicate that ERβ target genes are enriched in genes that regulate cell death/survival, cell movement, cell development, growth and proliferation, as well as genes involved in the Wnt/β-catenin and G1/S cell cycle phase checkpoint pathways [27–30]. Furthermore, the exact role of ERβ, especially when expressed alone is not well studied. The first priority is to identity new prognostic and therapeutic targets that can identity and treat TNBC. We predict that ERβ may be such a target in TNBC.

Emerging evidence indicates that metabolic factors, such as insulin-like growth factor (IGF-1 and IGF-2) pathways enhance the progression of BC [31–37]. IGF-2 occurs in two forms, precursor (pIGF-2) and mature (mIGF-2) and plays a role in BC proliferation and inhibition of apoptosis [38–40]. Under normal conditions IGF-2 is bound and sequestered [41–44], but overexpression of IGF-2 is associated with increased BC formation [43, 45]. Most human cancers over express IGF-1 receptor (IGF-1R) and IGF-2 is a known ligand for the respective receptor and can bind to IGF-2 receptor (IGF-2R), which sequesters IGF-2 [31–33, 35, 40]. Disparities with IGF-2 expression is also apparent, with AA having higher expression when compared to CA and could contribute to clinical outcome [46]. IGF-2 is expressed both in the BC stromal and epithelial compartments, and corresponds with both stromal and epithelial BC cell proliferation [33] [47–50]. ER signaling pathways are reported to intersect with IGF pathways, with estrogen promoting increased BC production of IGF2 [32]. In this study, we predict ERβ and/or IGF-2 may be such targets in TNBC.

Here, we report our findings on the importance of ERβ expression in TNBC progression. We investigated ERβ expression in different patient subtypes of breast cancer along with a panel of TNBC cell lines. We performed immunoblotting, quantitative-real-time PCR (qPCR), cell proliferation, migration/invasion, enzyme-linked immunosorbent assay (ELISA), and apoptosis studies on cell lines when ERβ is activated and/or inhibited. ERβ activation or inhibition resulted in significant differences in protein expression, migration/invasion, IGF2 expression, and apoptosis. This study also identified that the activation of ERβ activates the IR/IGF-1R signaling pathway via IGF2. We also show that activation of ERβ activated the MAPK signaling pathway.

## RESULTS

### ERβ is associated with decrease in relapse-free survival in TNBC

**Figure 1:**
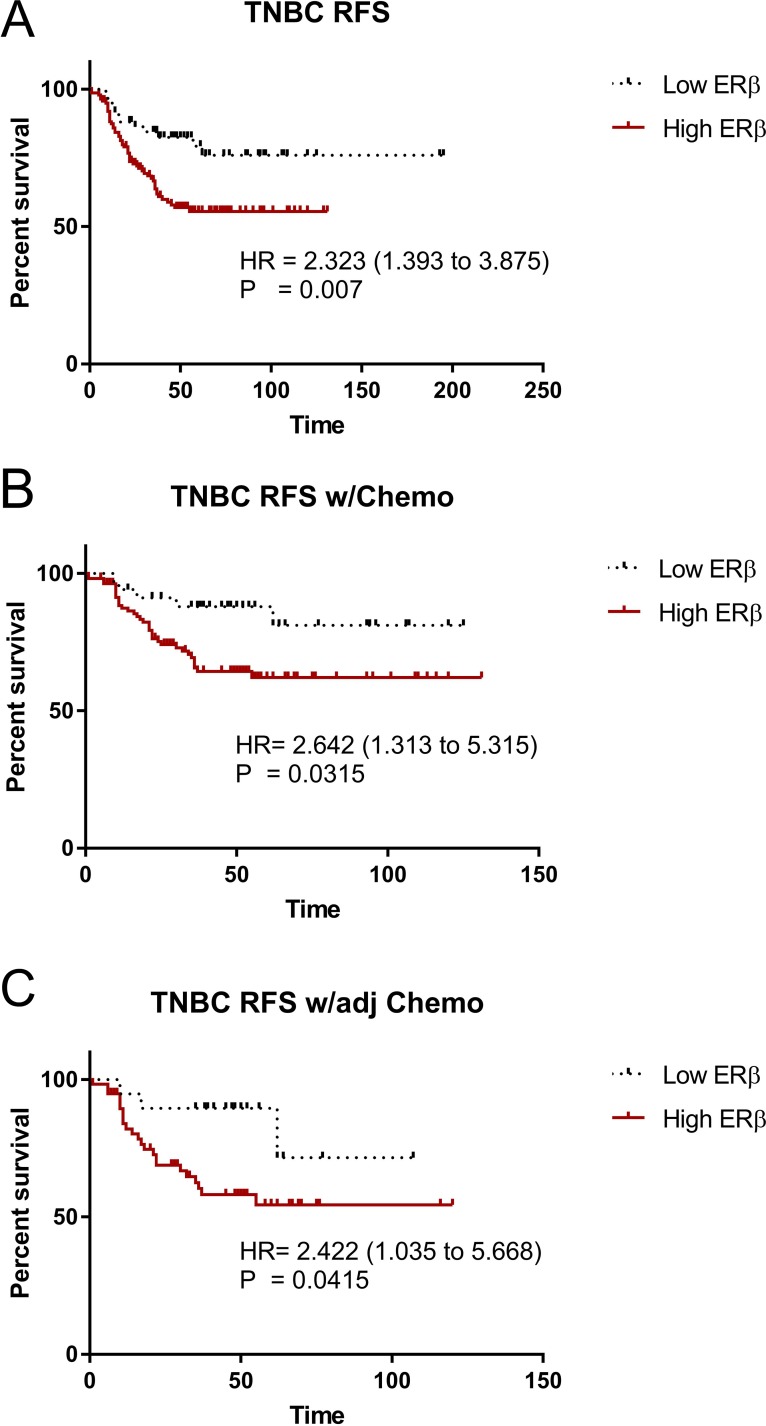
ERβ is associated with decrease relapse free survival in TNBC mRNA level of ERβ in TNBC was obtained from a public database, KMPlot. Kaplan–Meier survival analysis was used to assess the association between ERβ level and relapse-free survival (RFS). **(A)** RFS in all TNBC between Low ERβ and High ERβ groups, **(B)** RFS in TNBC underwent chemotherapy (Chemo) between Low ERβ and High ERβ groups, and **(C)** RFS in TNBC with adjuvant chemotherapy (adj Chemo) between Low ERβ and High ERβ groups. Log-rank test was used to assess the statistical significance between the groups and P<0.05 was considered as significance.

The roles of ERβ in breast cancer, let alone TNBC development and progression are not clearly elucidated. A database was established using mRNA expression data downloaded from gene expression omnibus (GEO) (http://www.ncbi.nlm.nih.gov/geo/), the cancer genome Atlas (TCGA) (http://cancergenome.nih.gov/), European genome-phenome archive (EGA) (https://ega.crg.eu/), and PubMed (http://www.pubmed.com) repositories to identify datasets with published mRNA expression and clinical data. Kaplan–Meier survival analysis was performed to validate the prognostic value. We determined the relapse-free survival (RFS) status between ERβ and TNBC, using the public database KMPlot [51, 52], which consisted of 3,951 breast cancer patients. KMPlot were used because it is a database integrating mRNA expression data and clinical information derived from four independent datasets of breast cancer. It is an easy-to-use bioinformatic tool capable of performing survival analyses (Months) for the identification and validation of prognostic mRNAs in breast cancer, and it allows for a real-time analysis to evaluate the prognostic value of those mRNAs in breast cancer. When accounted for TNBC patients only (restricted analysis to subtype hormone receptor negative: ERα-, PR-, HER2-), high ERβ expression is significantly correlated with lower overall RFS (P = 0.007) (Figure 1A). When patients were given chemotherapy as a whole (Figure 1B) or adjuvant chemotherapy (Figure 1C), high ERβ expression is significantly correlated with lower relapse free survival (RFS) (P = 0.0315 and 0.0415 respectively) and a trend with high ERβ expression is seen to coincide with lower distance metastasis free survival. These results suggest that high ERβ expression in TNBC patients might play a role in the progression of TNBC.

### ERβ expression in breast cancer cell lines

**Figure 2:**
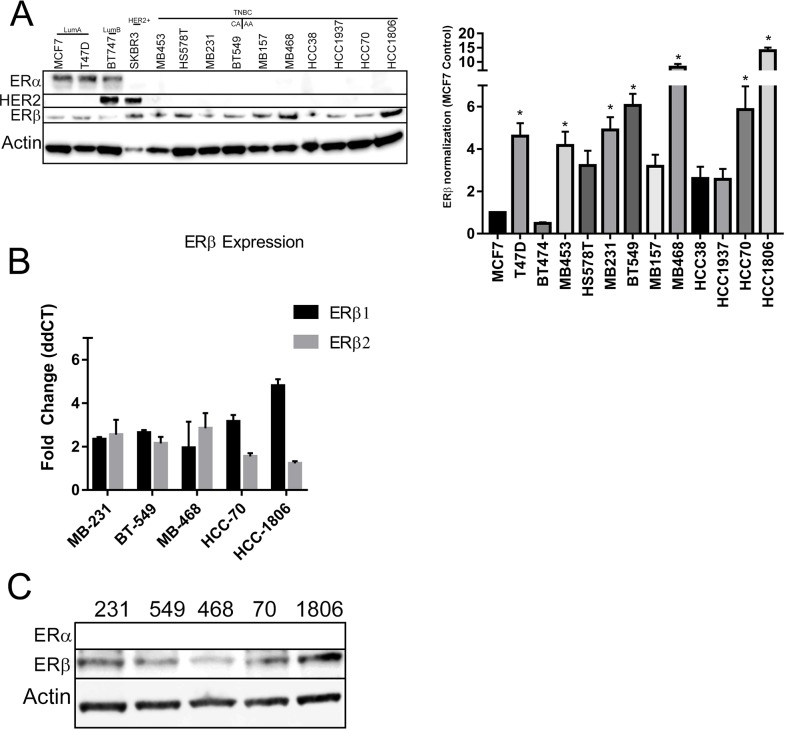
ERβ is expressed in TNBC cell lines **(A)** The indicated panel of breast cancer cell were maintained in growth media with 10% FBS and protein was extracted until the cells reach to 80% confluence. Western blot analyses were performed with ERα, ERβ1 and HER2 antibodies and β-actin antibody was used for loading control **(A-right)**. The western blot of ERβ1 was repeated 3 times and the bands intensities were adjusted to β-actin. The bar graph **(A-left)** showed the mean intensities from three independent bots and SD for the indicated cell lines. “^*^” indicated p<0.05 compared to the intensity from MCF-7 cells and determined by one-way ANOVA. **(B)** The indicated TNBC cells were grown in phenol red free media with 5% charcoal treated FBS until 80% confluence and RNA was extracted. RT-qPCR was performed with ERβ1 and ERβ2 primers and adjusted to GAPDH. The bar graph presented as fold changed of ERβ1 and ERβ2 levels in the indicated TNBC cells compared to MCF-7 cells. Each bar indicated mean±SD from three experiments. **(C)** The indicated TNBC cells were grown in phenol red media with 5% charcoal treated FBS until 80% confluence and protein was extracted. Western blot analyses with ERα and ERβ1 antibodies were performed and β-actin was used for loading control.

We assessed ERβ expression in a panel of several breast cancer cell lines from our lab. The cell lines we have represent the major subtypes of breast cancer: Luminal A (MCF7, T47D), Luminal B (BT474), HER2+ (SKBR3), and TNBC (BT549, HCC38, HCC70, HCC1806, HCC1937, HS578T, MBA157, MBA231, MBA453, MBA468). Luminal A cell lines will were taken as controls. Under normal growth conditions (10% FBS with phenol red), when compared to luminal A cell lines, all TNBC cell lines had increased expression of ERβ (Figure 2A right). Quantification of the bands are shown in Figure 2A left. Quantification of cellular lines were used to select 5 TNBC cell lines that represents African-American ethnicity (HCC70, HCC1806, MBA468) and Caucasian ethnicity (MBA231, BT549) for further studies. Selected cell lines were then analyzed for ERβ mRNA expression under 2% charcoal dextran stripped FBS in phenol red free media. Our data showed that all of the TNBC cells have at least a two fold increase in ERβ mRNA expression (Figure 2B) when compared to MCF7 cells. The respective cell lysates were subjected to gel electrophoresis and immunoblotting with ERβ and ERα antibodies. Results are shown in Figure 2C. TNBC cells show higher ERβ gene and protein expression.

### ERβ activation increases migration and invasion in TNBC cells

**Figure 3:**
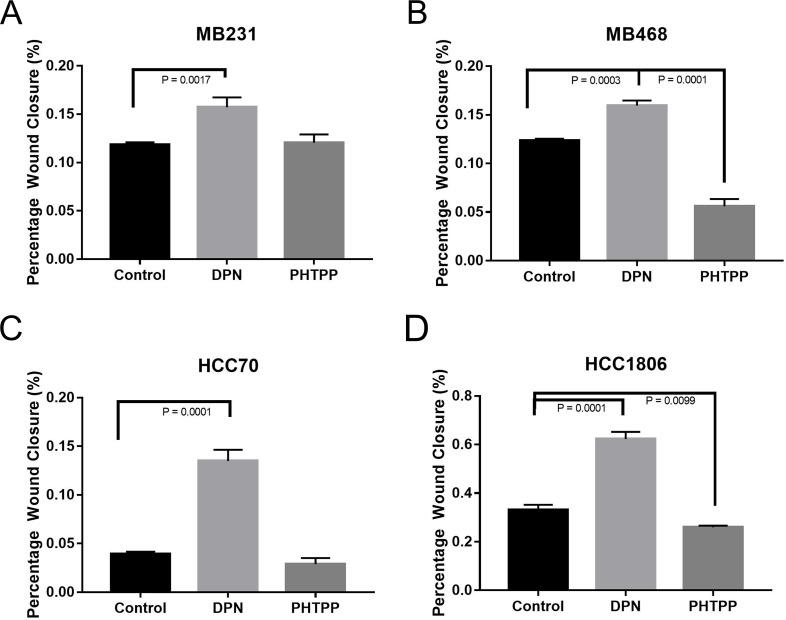
Activation of ERβ increases migration of TNBC cells The cells were seeded in 8.5cm3 dishes with a 2 well silicone insert and treated with or without DPN or PHTPP for 24 hrs as described in methods. The wound closure was monitored and images were taken and analyzed from three different rears for each conditions. The bar graphs for MB231 **(A)**, MB468 **(B)**, HCC70 **(C)** and HCC1806 **(D)** presented as percentage wound closures (mean ±SD) for each conditions. P-values between the indicated conditions were determined by one-way ANOVA.

To determine the effect of ERβ activation on cell migration, a wound healing assay on the TNBC cell lines were performed. TNBC cell lines were treated with known estrogen receptor agonist and/or antagonist, 20nM Diarylpropionitrile (DPN, ERβ agonist) and 100nM 4-[2-phenyl-5,7-bis(trifluoromethyl)pyrazolo[1,5-a]-pyrimidin-3-yl]phenol (PHTPP, ERβ antagonist) alone [53, 54]. TNBC cells were grown in 2-well inserts that gave a uniform scratch of 500μm in either control media (2% charcoal stripped FBS is phenol red free media), 20μM ERβ agonist (DPN), or 100μM ERβ antagonist (PHTPP). Images captured at 0, 4, 8, 12, and 24h after incubation using phase-contrast microscope. The rate of migration was measured by quantifying the total distance that the cells moved from the edge of the scratch toward the center of the scratch at 24h when compared to 0h. Activation of ERβ by DPN resulted in a significant increase in would closure in our TNBC cell lines (Figure 3A-3D). PHTPP treatment resulted in a significant decrease in wound closure in our MB468 (Figure 3D) and HCC1806 (Figure 3F) when compared to control. These results indicated that ERβ activation in TNBC cells results in increased cell migration.

**Figure 4:**
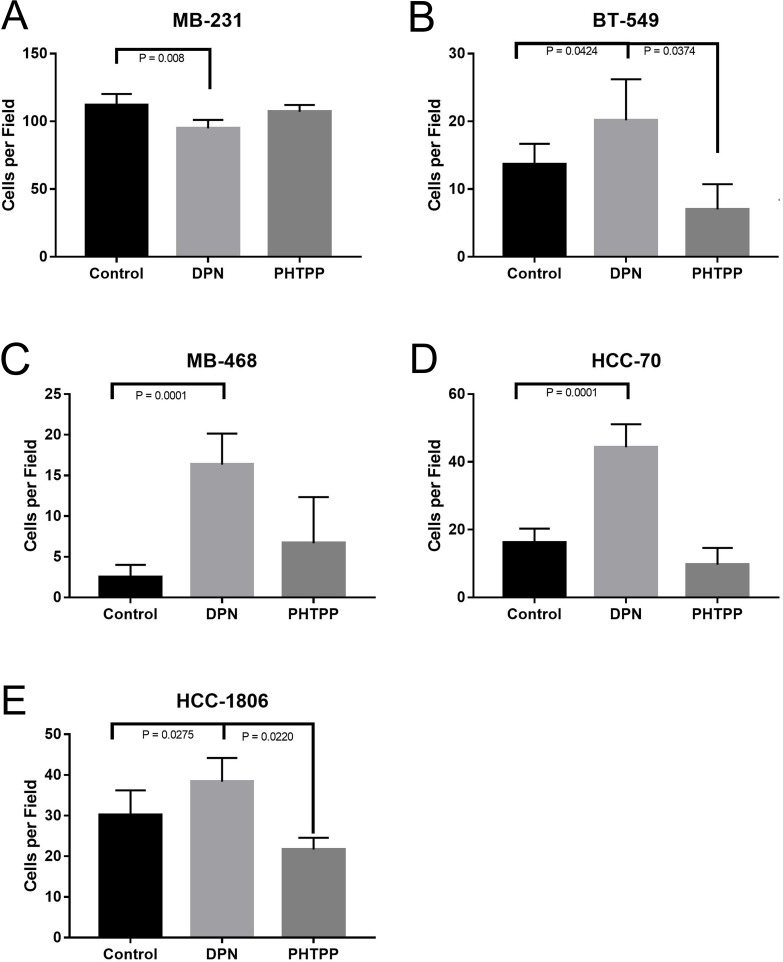
Activation of ERβ increases invasion of TNBC cells The cellswere plated in top chamber of matrigel invasion chamber in phenol red free media with or without DPN or PHTPP and bottom chamber was filled with medium containing CSFBS as described in methods. After 24 hrs number of cell invasion was assessed. The total numbers of invaded cells were counted from 4 independent areas for each conditions and all experiments were independently repeated three times. The bar graphs for MB231 **(A)** BT549 **(B)** MB468 **(C)** and HCC70 **(D)**, HCC-1806 **(E)** showed invaded cell numbers (mean ±SD) under each conditions. P-values between the indicated conditions were determined by one-way ANOVA.

We then wanted to investigate what role activation of ERβ on cellular invasion of TNBC cells. To determine the effect that ERβ may have on invasion, TNBC cells were grown in the Corning BioCoat Matrigel Invasion Chamber. Cells were treated with either DPN or PHTPP in the upper-chamber for 24 hours, and the cell invasion was measured. Cells were fixed, stained with crystal violet, and quantification of invaded cells was done. Treatment of DPN significantly increased invasion in all cell lines (P < 0.042) (Figure 4A-4E), except MB231 where we saw a significant decrease in invasion when treated with DPN (Figure 4B), and, similarly, the treatment with PHTPP significantly decreased cellular invasion of BT549 and HCC1806, respectively (P < 0.04, Figure 4B, 4E).

### ERβ activation increases proliferation in TNBC cells

**Figure 5:**
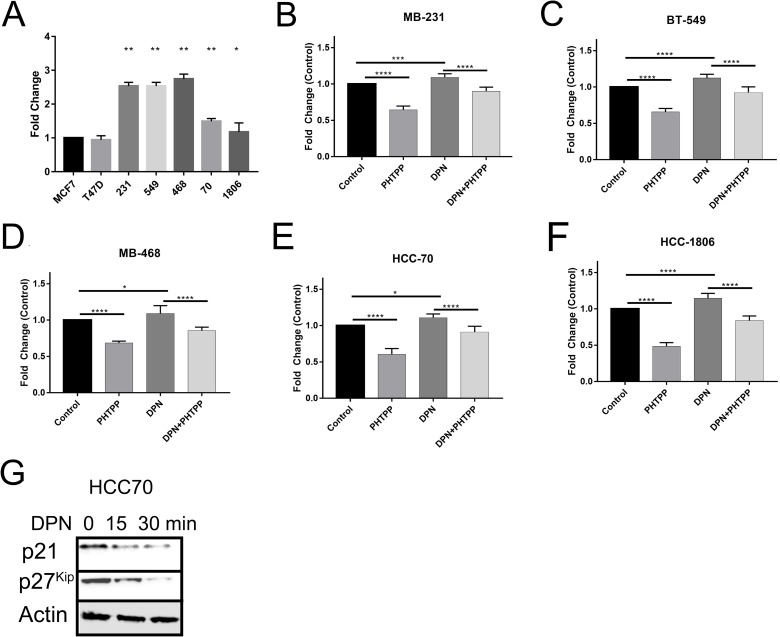
Activation of ERβ increases cell proliferation The cells were treated with or without DPN, PHTPP, or DPN+PHTPP for 3 days and cell proliferation was measured by The CellTiter-Glo® Luminescent Cell Viability Assay as described in methods. Proliferation rate of different TNBC cells without treatment **(A)**, MB231 **(B)**, BT549 **(C)** MB468 **(D)** HCC70 **(E)** and HCC1806 **(F)** cells with and without treated with DPN, PHTPP and DPN+PHTPP were determined by compared to MCF7 cells. The bar graphs indicated the mean fold-changes with SD from 3 independent measurements and each measurements contained 6 repeated values for each condition. The p-values were determined by One-Way ANOVA (^*^P < 0.01, ^**^P < 0.001, ^***^P < 0.0009, ^****^P < 0.0001). **(G)** HCC70 cells were treated with DNP for the indicated time and protein was extracted. Western blot analysis was performed with p21 and p27^kip^ antibodies and β-actin was used as loading control.

The data presented here shows that activation of ERβ is involved in the migration and invasion of TNBC cells. We then wanted to investigate the effect of ERβ activation on cellular proliferation. To determine the effect of the activation of ERβ on TNBC cells we treated TNBC cells with DPN, PHTPP, or both over a 3 day period. Cell proliferation was measured by CellTiter-Glo. Quantification of cell proliferation was then measured by relative luciferase units (RLU), and then normalized to control to get fold change. Proliferation rate of TNBC cells were compared to control (Luminal A – MCF7 cells). Under normal conditions over a three day period we saw that all TNBC cell lines had a significant increase, at least a 1.5fold, in cellular proliferation when compared to control MCF7 cells (Figure 5A). We saw the highest proliferation rates in mesenchymal-like TNBC cells (MB231/BT549) vs basal-like subtype except for MB-468 cells. Next, we determined the proliferation rate over a three day period, when treated with either DPN and/or PHTPP. We saw that treatment with PHTPP significantly decreased cellular proliferation over a three day period and DPN treatment increased cellular proliferation (Figure 5B-5F). When TNBC cell lines were treated with both agonist and antagonist returned proliferation back to control levels. This increase in cellular proliferation could be due to ERβ down regulation of cell cycle inhibitors p21 and p27^kip^ (Figure 5I). This data highlights that ERβ promotes cell proliferation when expressed in TNBC.

### ERβ activation upregulates IGF2 and the IR

**Figure 6:**
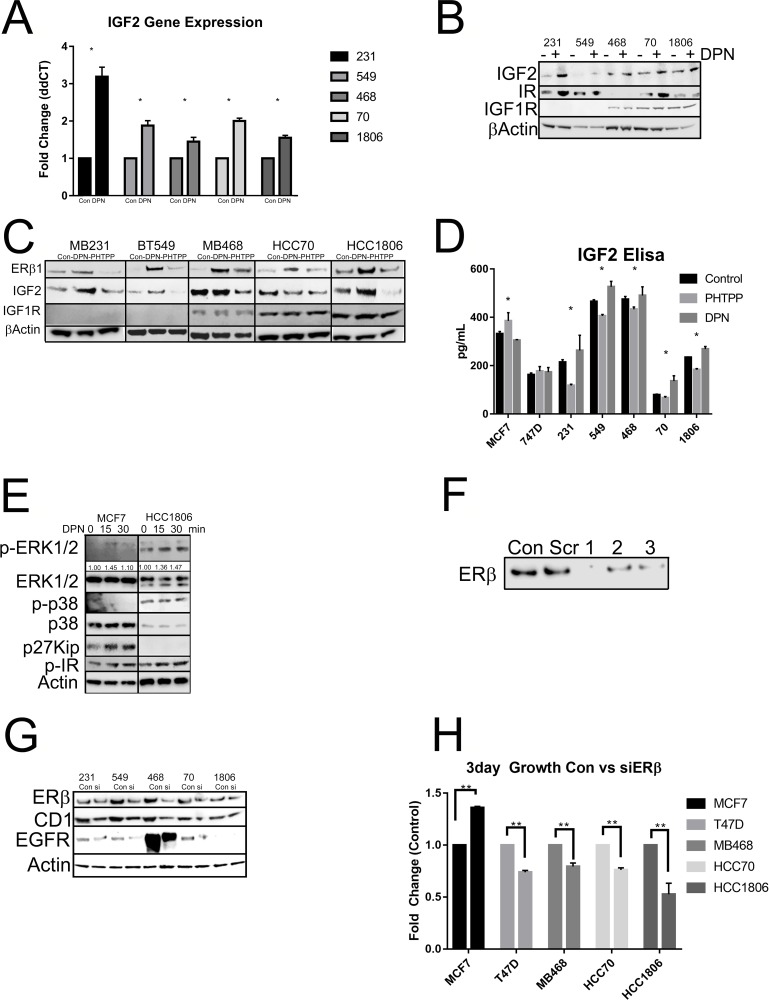
Activation of ERβ upregulates IGF2 and activates the IR/IGF1R/MAPK pathways **(A)** The indicated cells were treated with or without DPN. mRNA level of IGF2 was determined by RT-qPCR and adjusted for GAPDH. The bar graph presented fold changes of IGF2 level in cells treated with DPN compared to non-treated cells (control). Each bar indicated mean ±SD from 3 measurements and “^*^” indicated p<0.05 determined by one-way ANOVA. **(B)** Total protein was extracted from the indicated cells treated with or without DPN. Western blot analysis was performed with antibodies for IGF2, insulin receptor (IR) and IGFIR. β-actin was used for loading control. **(C)** The cells were treated with or without DPN, PHTPP or DPN+PHTPP as descripted in methods and total protein was extracted. Protein levels of ERβ1, IGF2 and IGFIR were determined by Western Blot analysis and β-actin was used as loading control. **(D)** The cells were maintained in serum free media and treated with or without DPN or PHTPP. Secreted protein level of IGF2 in the media was measured by IGF2 ELISA kit according to manufacture instruction as described in methods. The bars indicated mean level of IGF2 with SD from duplicated measurements and “^*^” indicated P<0.05 for the differences in level of IGF2 between the indicated cells and MCF7 cells determined by one-way ANOVA. **(E)** The cells were treated with DPN from 0 to 30 min and protein was extracted. Western blot analysis was performed with antibodies for P-ERK1/2, ERK1/2, P-p38, p38 and p27^kip^. β-actin was used as loading control. The level of P-ERK1/2 was further quantified with β-actin and showed under membrane of P-ERK1/2. **(F)** T47D cells were treated with 3 different siRNA sequences of ERβ and ERβ1 expression was determined Western Blot analysis with antibody for ERβ1 was used to assess efficiency of the siRNAs. **(G)** The cells were treated with pooled siRNA sequences for ERβ and the level of ERβ was determined by Western blot. The protein levels of cyclin D1 (CD1) and EGFR were also determined by Western blot analysis. β-actin was used as loading control. **(H)** The cells were treated with or without siRNA for ERβ and cell proliferation was determined by The CellTiter-Glo® Luminescent Cell Viability Assay as described in methods. The bars indicated fold changes (mena ±SD) of cells treated with siRNA compared to cells without treating with siRNA (control). “^**^” indicated P<0.001 between the indicated groups determined by one-way ANOVA.

Since previous data has shown that activation of ERβ resulted in increased proliferation of TNBC cell lines, we then wanted to determine by what mechanism is ERβ regulating cell growth. When we treated our cell lines with DPN and/or PHTPP we saw an upregulation in IGF2 mRNA (Figure 6A) and IGF2 and insulin receptor (IR) protein expression by DPN (Figure 6B). TNBC cells treated with PHTPP resulted in decreased IGF2 protein expression (Figure 6C). When TNBC cell lines were treated with DPN or PHTPP, a significant increase in IGF2 secretion by DPN and a decrease in IGF2 secretion by PHTPP in TNBC cell lines (Figure 6D). We then looked at what pathways were activated by ERβ. The data showed that the MAPK pathway and the IR pathway were activated in HCC1806 when compared to MCF7 cells and growth inhibition by DPN seen in MCF7 was due to the inhibition of the cell cycle through p27^kip^ (Figure 6E). This data highlights that activation of ERβ increases IGF2 secretion that can then act on the IR/IGF1R signaling pathway to control cell growth.

### Knockdown of ERβ results in decreased proliferation

**Figure 7:**
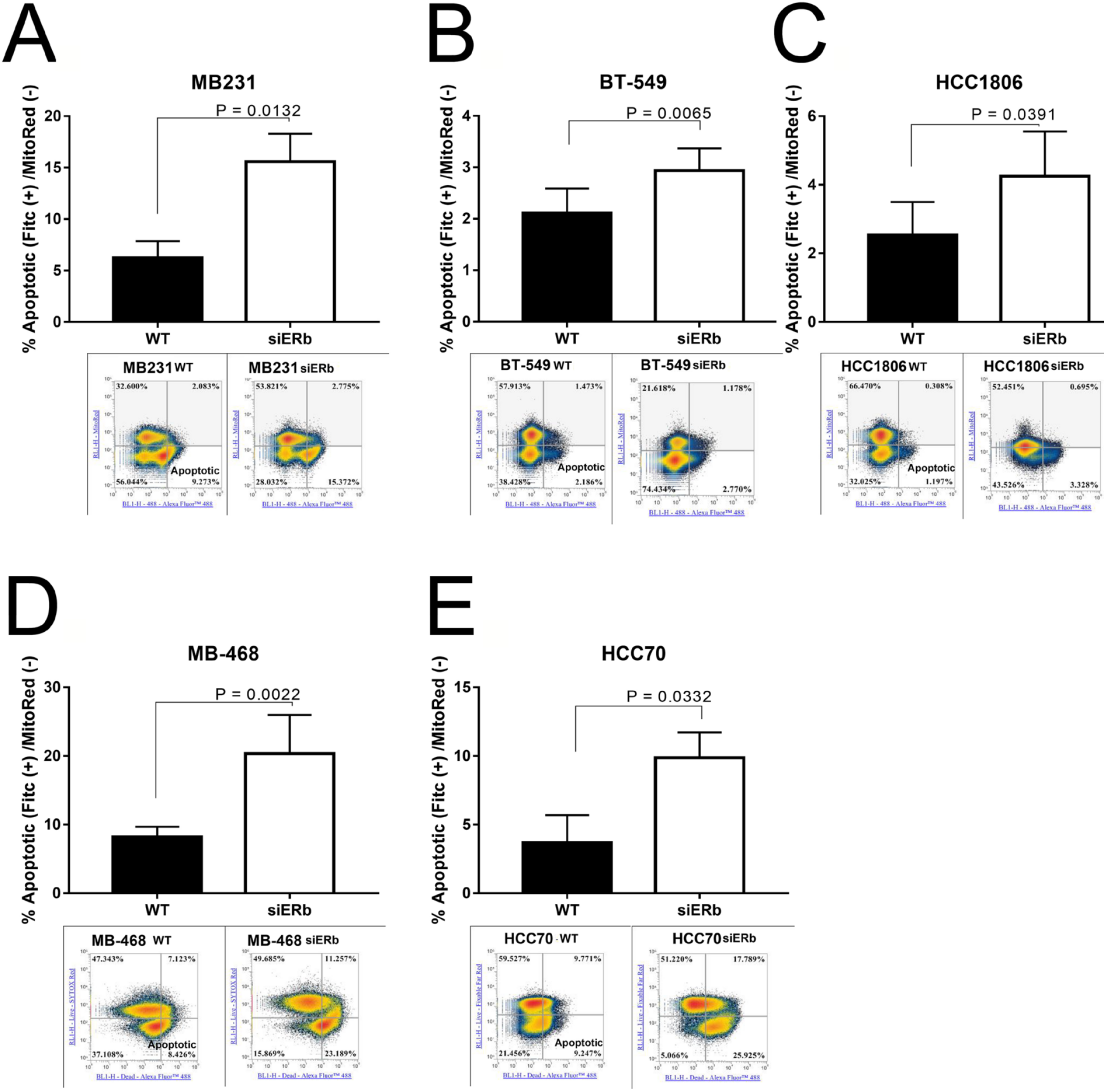
Knockdown of ERβ results in increased apoptosis TNBC cells were knockdown for ERβ and then subjected to flow cytometer as described in methods Representative images of flow cytometry data were presented. Percentage of apoptotic cells in **(A)** MB231, **(B)** BT-549, **(C)** MB-468, **(D)** HCC-70, and **(E)** HCC-1806 were showed in bar graphs as mean ±SD. The statistical differences between the indicated groups were determined by paired *t*-test.

We then wanted to determine what role knockdown of ERβ had on cellular proliferation. We used scrambled and three pools of siRNA towards ERβ. Figure 6F shows the knockdown efficiency with the scramble and three different pools of siRNA. We saw that each siRNA was able to knockdown ERβ in T47D cells (Figure 6C). The pooled siRNA were transfected in our TNBC cell lines and through western blot determined the effect of ERβ knockdown on protein expression. Knockdown of ERβ was able to downregulate Cyclin D1 (CD1), EGFR, and ERβ (Figure 6G). We then looked at cellular proliferation. Knockdown of ERβ had a significant decrease in cellular proliferation (Figure 6H). We then wanted to determine what role ERβ plays in apoptosis. Apoptosis was determined by measuring the uptake of annexin in control cells (untreated), compared to cells with ERβ knockdown. The stained cells were analyzed by flow cytometry. Images represent 4 independent flow cytometer runs. As shown in Figure 7A-7E, a significant increase in apoptosis was seen in ERβ knocked down cells (Figure 7).

### ERβ expression in clinical samples using tissue microarray

**Figure 8:**
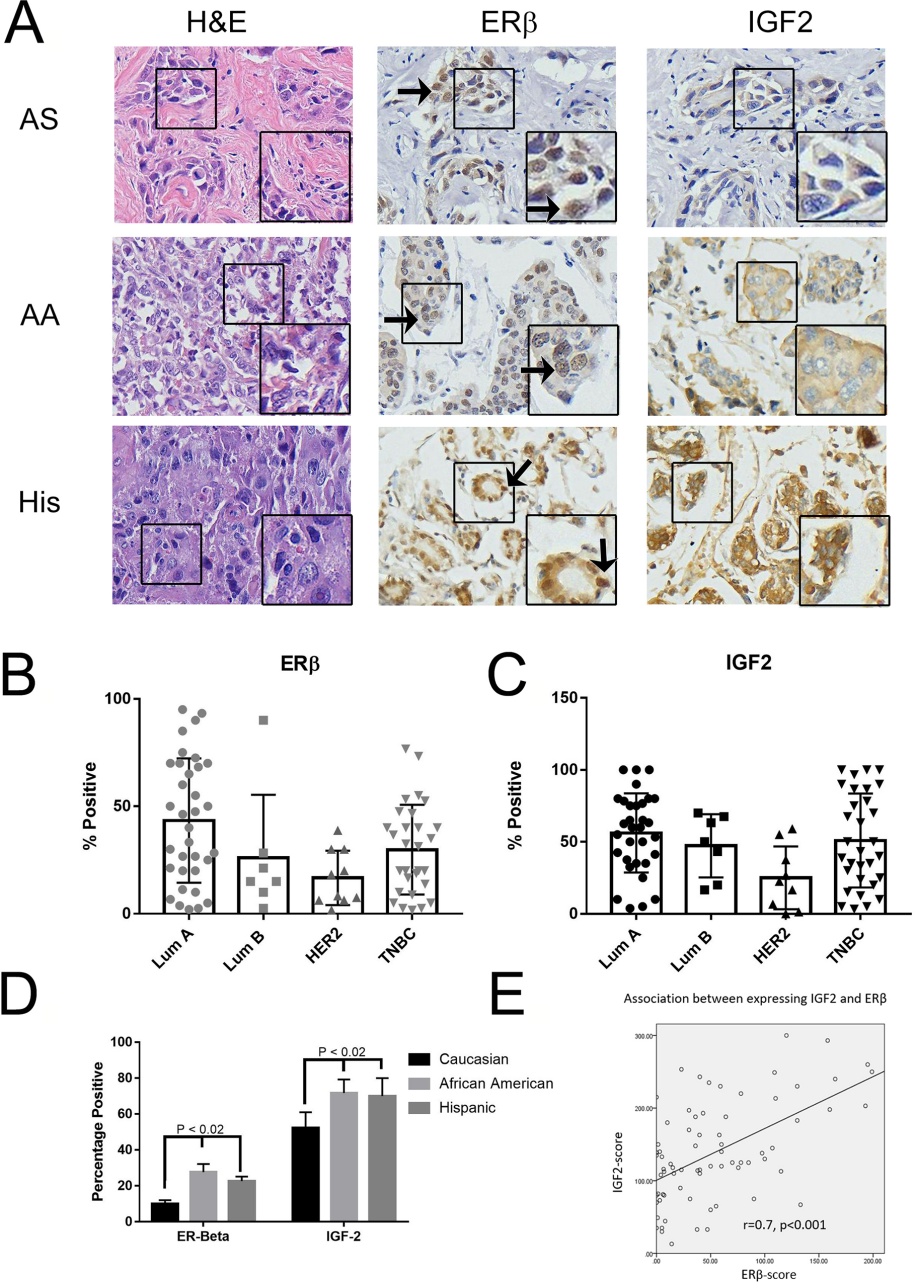
ERβ is expressed in breast cancer patients' tissue microarray **(A)** Immunohistochemical (IHC) analysis was used to determine ERβ1 and IGF2 expression in breast cancer primary tissues as described in methods. Represented images from IHC of H&E, ERβ1 and IGF2 in TNBC from Asian (AS), African American (AA), and Hispanic/Latina (His) patients were shown. **(B and C)** Bar graphs showed quantified IHC data of ERβ1 **(B)** and IGF2 **(C)** expression in different subtypes of breast cancer. The bars indicated percentage of positive staining for ERβ1 or IGF2 (mean ±SD) in the indicated subtype of breast cancers. **(D)** IHC data of ERβ1 was quantified and the bars indicated percentage positive expression of ERβ1 in the indicated racial ethnicities. **(E)** The correlation between expressing ERβ and IGF2 in patients was evaluated by Bivariate Correlations with two tailed test, performed with SPSS v.22.

We have seen that through the public database that high ERβ expression is correlated with lower RFS, and in TNBC cell lines that activation of ERβ resulted in increased cellular proliferation, migration, and invasion. We then wanted to see ERβ expression in TNBC across ethnicity. The make-up of our patient population is located in Table 1. As illustrated in Figure 8A, we had archival TNBC tissue specimens from Asian (AS, commercially purchased from US Biomax), African-American (AA), Hispanic (His), and Caucasian (CA) samples from the patient cohort in South Los Angeles. When compared to AS, AA and His patients had higher ERβ and IGF2 expression (Figure 8A and Table 2). When separated by tumor type, both ERβ (Figure 8B) and IGF2 (Figure 8C) are expressed in similar levels within TNBC and Lum A cancers. When separated by race, looking at TNBC only, we see that ERβ and IGF2 are expressed significantly higher in AA and His when compared to CA (P < 0.02) (Figure 8D). We then looked at the association between ERβ and IGF2 and saw that, within TNBC, IGF2 and ERβ expression correlated with each other (Figure 8E) and that IGF2 was associated with increased ERβ expression (Table 3). This data highlights that African American and Hispanic patients with TNBC had higher ERβ and IGF2 expression. This co-expression may result in decreased overall survival of TNBC patients.

**Table 1:** Subjects in CDU TMA

	N (%)
Total	204
**Ethnicity**	
African American	50 (24.5)
Hispanic	28 (13.7)
Asian	126 (61.8)
**Age (yr)**	
<50	90 (44.1)
≥50	114 (55.9)
**ERα/PR status**	
Positive	130 (63.7)
Negative	74 (36.3)
**HER2 status**	
Positive (IHC 3+/FISH amplified)	52 (25.5)
Negative	152 (74.5)
**Subtype (by receptors)**	
ERα/PR+/HER2-	104 (51.0)
HER2+	52 (25.5)
TNBC	48 (23.5)
**Tumor size**	
≤ 2cm	45 (22.1)
2cm – 5cm	115 (56.4)
> 5cm	32 (15.7)
Unknown	12 (5.9)
**Node**	
Negative	68 (33.3)
Positive	127 (62.3)
Unknown	9 (4.4)
**Stage**	
0-II	134 (65.7)
III-IV	66 (32.4)
unknown	4 (2.0)
**ERβ (nuclear)**	
Positive	121 (59.3)
Negative	83 (40.7)
**IGF2**	
IGF2-positive	41 (19.7)
IGF2-negative	159 (76.4)
missing	8 (3.8)

**Table 2:** ERβ and IGF2 status by ethnicity, age, tumor pathology and stage

	N (%)						
Total	204	ERβ-positive	ERβ-negative	p-Value	IGF2-Positive	IGF2-negative	P-Value
**Ethnicity**							
African American	50 (24.5)	36 (72.0)	14 (28.0)		26 (55.3)	21 (44.7)	
Hispanic	28 (13.7)	18 (64.3)	10 (35.7)		11 (40.7)	16 (59.3)	
Asian	126 (61.8)	67 (53.2)	59 (46.8)	**0.019**	4 (3.2)	122 (96.8)	**<0.001**
**Age (yr)**							
<50	90 (44.1)	52 (57.8)	38 (42.2)		21 (23.9)	67 (76.1)	
≥50	114 (55.9)	69 (60.5)	45 (39.5)	0.774	20 (17.9)	92 (82.1)	0.378
**ERα/PR status**							
Positive	130 (63.7)	81 (62.3)	49 (37.7)		26 (20.3)	102 (79.7)	
Negative	74 (36.3)	40 (54.1)	34 (45.9)	0.3	15 (20.8)	57 (79.2)	1.0
**HER2 status**							
Positive	52 (25.5)	26 (50.0)	26 (50.0)		7 (14.0)	43 (86.0)	
Negative	152 (74.5)	95 (62.5)	57 (37.5)	0.141	34 (22.7)	116 (77.3)	0.228
**Subtype**							
ERα/PR+/HER2-	104 (51.0)	69 (66.3)	35 (33.7)		23 (22.3)	80 (77.7)	
HER2+	52 (25.5)	26 (50.0)	26 (50.0)		7 (14.0)	43 (86.0)	
TNBC	48 (23.5)	26 (54.2)	22 (45.8)	0.089	11 (23.4)	36 (76.6)	0.912
**Tumor size**							
≤ 2cm	45 (22.1)	29 (64.4)	16 (35.6)		12 (27.3)	32 (72.7)	
2cm – 5cm	115 (56.4)	63 (54.8)	52 (45.2)		11 (9.7)	102 (90.3)	
> 5cm	32 (15.7)	23 (71.9)	9 (28.1)	0.677	14 (43.8)	18 (56.3)	0.208
**Node**							
Negative	68 (33.3)	44 (64.7)	24 (35.3)		15 (22.4)	52 (77.6)	
Positive	127 (62.3)	74 (58.3)	53 (41.7)	0.443	23 (18.4)	102 (81.6)	0.570
**Stage**							
0-II	134 (65.7)	79 (59.0)	55 (41.0)		27 (20.6)	104 (79.4)	
III-IV	66 (32.4)	41 (62.1)	25 (37.9)	0.759	13 (19.7)	53 (80.3)	1.0

**Table 3:** Association between expressing IGF2 and ERβ

	IGF2 Status		P-Value
	High	Low	
ERβ-positive	32 (78%)	87 (54.7)	
ERβ-negative	9 (22.0)	72 (45.3)	0.07

## DISCUSSION

Breast cancer patients who are presented with TNBC are frequently diagnosed at an advance stage of the disease, with high histologic grade, and are associated with a significantly higher probability of relapse and metastasis. This clinical diagnosis is as a health disparity, and currently, the most common treatment is systemic chemotherapy [3–11]. Discovery and higher expression of ERβ made it a logical therapeutic target because of its expression in TNBC and its structural similarity to ERα [13–15], however there have been conflicting results, some suggesting that ERβ is of favorable prognostic value [20, 55, 56], no prognostic value [57], or worse prognosis [16, 30, 58]. Some of the discrepancies can also be due to the fact that TNBC is a heterogeneous disease, and most studies that look at TNBC used the more accessible TNBC cell lines (MB231 and Hs578T) and did not account for the heterogeneity of TNBC. There are four subclasses of TNBCs [59] such as basal-like (BL) [7], mesenchymal (MS) [60], immune-enriched (IM) [7, 61], and the luminal AR subtype (LAR) [61]. Others have suggested that the discrepancy may be due to a lack of standardized detection methods, poorly validated antibodies, inconsistent cutoffs for defining ERβ positive cancers via IHC, and variable tissue preparation and processing methods [62, 63]. To address these discrepancies we used validated TNBC specimens (Negative for ERα. PR, and HER2 overexpression). We used validated ERβ antibodies [30, 63, 64], TNBC subtypes (BL1/2, MS, and MSL), and different ethnicities (AA: MB468 HCC-70/1806 CA: MCF7 T-47D, MB-231, BT-549).

Here we examined the role ERβ in disease progression, proliferation, invasion, and migration of TNBC. Our findings show that contrary to previous studies, ERβ activation is pro-tumorigenic *in vitro*. The relationship between the expression of ERβ and breast cancer clinical progression has been ambiguous, in the publically available data. TNBCs reviewed for our study have shown that high ERβ expression results in significantly lower RFS with or without chemotherapy. We have also shown that in TNBC cell lines that activation of ERβ results in increased secretion of IGF2 which can bind to IR/IGF1R, and activate growth promoting capabilities. Recent studies have shown that targeting the IGF1R pathway could be a therapeutic target [65, 66]. These results and the previous data suggest that endocrine therapy may be beneficial for improving TNBC outcomes.

ERβ has been suggested to control a myriad of functions in BC. Here we have shown that in most TNBC cells, activation of ERβ by DPN significantly increased cell invasion and migration, especially in cells that aren't highly invasive (MB468, HCC-70/1806). However, in MB-231 cells, DPN caused significant decrease in cellular invasion. This discrepancy could be due to the that fact that a recent study [67] had shown that MB-231 cells express androgen receptor (AR) and that in AR positive TNBC cells, ERβ decreases cellular invasion and migration. Another discrepancy could be due to the fact that these authors used an over-expressing ERβ construct, used FBS and phenol red containing media to determine invasion instead of using hormonal free FBS/phenol red free media. In our study we used a specific agonist to activate ERβ expression, DPN. Even though our results corroborated on the decreased invasion by the activation of ERβ in MB-231 cells, the inhibition of migration (wound closure) due to ERβ activation didn't agree. Also, we saw in another MS subtype, BT-549, that ERβ activation increased cellular invasion. This supports the notion that in specific subtypes of TNBC ERβ could have different effects.

In this study we also wanted to look at what pathways are activated by ERβ. In our ERα+ cell lines (MCF7, T47D) ERβ activation results in the increase expression of p21^WAF/CIP1^ and p27^kip^ indicating that in ERα+ cell lines, ERβ provides and anti-proliferative role and agrees with overall BC RFS and other studies showing the anti-proliferative role of ERβ in ERα+ tumors [14–16, 20, 68, 69]. Cyclin–CdK activity is regulated by phosphorylation events and cyclin kinase inhibitors, inhibit their activity. Our results showed that the upregulation of p21/27 might be one of the possible mechanisms of proliferation inhibition by ERβ in ERα+ tumors and the lack of activation of these inhibitors in TNBC. In TNBC, the activation of phospho-ERK1/2, p38, SAPK/JNK, and PI3K which are involved in the MAPK and PI3K signaling pathways. This supports previous studies that activation of ERβ resulted in the activation of MAPK/PI3K signaling [70, 71]. Knocked down (siRNA)/inhibited ERβ by a specific ERβ antagonist (PHTPP) saw a down regulation in Cyclin D1, EGFR, and MAPK/PI3K signaling, indicating ERβ roles in cell cycle and MAPK/PI3K signaling. We have also shown that down regulation of ERβ increase apoptosis in TNBC cell lines. Further studies are warranted to identify the precise mechanism by which ERβ overexpression can repress apoptosis in TNBC tumors.

In conclusion, the results of this study indicates that expression ERβ in TNBC patients is significantly associated with decreased RFS and RFS with/without chemotherapy. ERβ and IGF2 expression is positively correlated with each other, ERβ/IGF2 expression is increased in AA/HS women when compared to CA and AS. ERβ activation significantly increases invasion, migration, proliferation, and IGF2 secretion. When silencing ERβ or using a specific ERβ antagonist, we demonstrated that ERβ suppression resulted in decreased IGF2 secretion and proliferation. This suppression could possibly be due to the suppression of the MAPK/PI3K/AKT pathways and IGF2 activation on IR/IGF-1R. This work further highlights the roles ERβ plays in the heterogeneity of TNBC and can lead us to more promising therapeutics to specifically target ERβ or use combinational targets for ERβ, IR/IGF-1R, MAPK/PI3K/AKT pathway signaling.

## MATERIALS AND METHODS

### Patients

Patients were selected from an ongoing breast cancer study conducted in the Division of Cancer Research and Training at Charles R. Drew University of Medicine and Science in South Los Angeles. Women were informed and consented from Martin Luther King Ambulatory Care Center (MACC) between 1995 and 2007. This study was approved by the Charles R. Drew University of Science and Medicine Institutional Review Board and written informed consent was obtained from all participants (Approval 00-06-041-13). A total 1400 participants have been consented into the study, and 370 subjects have breast cancer confirmed by surgical biopsy/pathology and follow-up data. Characteristics of tissue microarray are found in Table 1. Charles R. Drew University breast cancer patient tissue microarray contains African-American (AA) and Hispanic (His) breast cancer tissue. Asian (AS) tissue microarray came from US Biomax.

### Definition of breast cancer subtypes

The receptor subtypes were categorized in the following manner: (a) Luminal A (LumA) (ER+ and/or PR+) and HER2-, (b) LumB (ER+ and/or PR+) and HER2+, (c) and HER2+, and (d) triple negative (TNBC) (ER−/PR−/HER2−) based on immunohistochemistry (IHC) analysis. The ER/PR, HER2, and Ki67 status were obtained from the patient's pathology reports. HR+ was defined as >5% nuclear positive for ER and/or PR in tumor cells. HER2+ was defined as HER2 3+ by IHC and/or more than 2.2 HER2 genes counted for every copy of chromosome 17 (HER2/CEP17 ratio) by FISH analysis. Ki67 Low was defined as <= 20% nuclear positive, and Ki67 High was defined as > 20% nuclear positive. Asian patient tissue microarray was obtained from US Bio Max, Inc (Rockville, MD). Staining and receptor status are located on manufactures web page, sample: HBre-Duc140Sur-01 (https://www.biomax.us/tissue-arrays/Breast/HBre-Duc140Sur-01).

### Cells, reagents, and antibodies

Triple negative breast cancer cells (TNBC; MDA-MB-231/468, BT-649,) and ER/PR positive breast cancer cells (MCF7 and T-47D) were cultured in DMEM/F12 (Gibco, Grand Island, NY), 10% fetal bovine serum (Gibco), and 1% penicillin/streptomycin (Gibco). Triple negative cell lines HCC 70/1806 were cultured in RPMI 1640 (Gibco), 10% FBS (Gibco), or 5% dextran-coated charcoal-stripped FBS (CSFBS) (GE Life Sciences Pittsburgh, PA), and 1% penicillin/streptomycin (Gibco). Primary antibodies against ERβ1 (clone PPG5/10) were purchased from BioRad(AbDSerotec) (Valencia, CA), Insulin Receptor (Abcam) IGF2 from ThermoFisher (Rockford, IL), CNND1, EGFR, ERα, Beta-Actin from Santa Cruz Biotechnology (Santa Cruz, CA), p-ERK1/2, p-p38, p27, p21 from Cell signaling (Beverly, MA). ERβ agonist, Diarylpropionitrile (DPN) and antagonist, 4-[2-phenyl-5,7-bis(trifluoromethyl)pyrazolo[1,5-a]-pyrimidin-3-yl]phenol (PHTPP) were purchased from Tocris.

### Cell migration test

TNBC cells were seeded in 8.5cm^3^ dishes and cultured in culture-insert 2 wells from Ibidi (Munich, Germany) to give a uniform scratch at 500μM. A line was created by removing the culture insert. Cells were then treated with DPN or PHTPP for 24hrs and pictures were taken at 0, 2, 4, 8, 12, and 24hr. The wound area was marked and cell migration into the scratched area was daily photographed using an inverted microscope.

### Cell invasion assay

One day prior to beginning the assay, TNBC cells were starved in a serum-free medium in top chamber of Corning BioCoat matrigel invasion chamber with 8.0mm PET membrane (Corning Corning, NY). Invasion assay was performed using the matrigel invasion assay kit. Briefly, top chamber was plated with 5 × 10^4^ cells in serum-free, phenol red free medium with either 20mM DPN or 100mM PHTPP. Bottom chamber was filled with medium containing CSFBS and incubated at 37°C with 5% CO2 for 24 hours. After the fixation of cells to the bottom chamber, the upper chamber was removed of all cells, and then coated with crystal violet for 15mins. Chamber was then wash and analyzed for cell invasion by the detection of crystal violet positive cells on the bottom chamber. The total numbers of invaded cells were counted by hand in 4 independent sections. All experiments were independently repeated at least three times.

### Apoptosis assay

Measurement of apoptosis was done by using Mitochondrial Membrane Potential Apoptosis Kit, with Mitotracker™ Red & Annexin V Alexa Fluor™ 488, for flow cytometry per manufacture protocol by Thermofisher. Cells were stained and then analyze the cells by flow cytometry, measuring the fluorescence emission at 530 nm and 585 nm. The cells should resolve into two principal populations: live cells with a low level of green fluorescence and high red fluorescence, and apoptotic cells with moderate level of green fluorescence and low red fluorescence by our Attune NxT Flow Cytometer.

### Transfection assay

ERβ si/shRNA was purchased from Santa Cruz Biotechnology. The transfection was performed on the Lonza 4D-Nucleofector™ System per manufacture protocol.

### Cell viability assay

Cell viability assay was performed using CellTiter-Glo (Promega) according to manufacturer's instructions. TNBC and control cells were seeded at 2,000 cells/well in a 96-well plate, grown over 3 and 5 days in the presence and absence of DMSO, DPN, and PHTPP. CellTiter-Glo measurements were taken at several time points to track cell survival.

### Quantitative-real-time PCR, western blot and IHC

For quantitative real-time PCR (qPCR) RNA was extracted with Trizol (Ambion) from 46 Surgical BPH and 53 Incidental BPH specimens. Subsequently, 500 ng RNA was reverse transcribed into cDNA using iScript cDNA Synthesis Kit (BioRad, Valencia, CA). qPCR was performed using IQ SYBR Green Supermix (BioRad, Hercules, CA) and results were analyzed using BioRad CFX manager software. All results were calculated using ΔΔCt analysis and normalized to GAPDH expression.

For Western blotting, protein was extracted with 2% SDS buffer and 25 μg protein was run on pre-made 4-12% polyacrylamide gels (Life Technologies). Primary antibodies were incubated in 5% BSA in TBST for 1 hour or overnight or at 4°C followed by incubation in secondary antibodies and development using ECL. Membranes were stripped and re-probed with an antibody against β-actin (Sigma).

Immunohistochemistry was performed as previously described [72] Briefly, 5 μm sections were de-waxed, rehydrated and endogenous peroxidases were blocked with hydrogen peroxide. Sections were then boiled in citrate and blocked in 5% serum for 1 hr. Primary antibodies were incubated overnight at 4°C at the following concentrations: ERb (1:100) and IGF2 (1:200). Biotinylated anti-mouse or -rabbit secondary antibodies (DAKO Carpentaria, CA) were incubated for 60 min at room temperature after slides were washed for 1 hr in PBS. Slides were incubated in ABC-HRP complex (Vector Laboratories Burlingame, CA) for 30 min. Bound antibodies were then visualized by incubation with 3,3′ diaminobenzidine tetrahydrochloride (liquid DAB, DAKO). Slides were then rinsed in tap water, counterstained with hematoxylin, and mounted.

### Statistical analysis

Log-rank (Mantel-Cox) test was used to determine overall survival and relapse free survival between High and low ER-β gene expression. Mann–Whitney tests were used for univariate comparisons of study characteristics between two groups. A two-sided P-value of 0.05 or less was considered statistically significant. A two-way ANOVA test was used for comparisons of characteristics. Correlation between IGF2 and ER-β was evaluated by the determination of the Spearman correlation coefficient (GraphPad Prism).

## Abbreviations

AA: African-American women
AR: androgen receptor
BC: Breast Cancer
CA: Caucasian American women
DPN: Diarylpropionitrile
ELISA: enzyme-linked immunosorbent assay
ER: Estrogen Receptor
ERα: ER-alpha
ERβ: ER-beta
HER2: Human Epidermal Growth Factor Receptor 2
HS: Hispanic women
IGF: insulin-like growth factor (IGF-1 and IGF-2)
IGF-1R: IGF-1 receptor
IGF2R: IGF-2 receptor
IHC: immunohistochemical
LAR: luminal AR subtype
mIGF2: mature IGF2
PHTPP: 4-[2-phenyl-5,7-bis(trifluoromethyl)pyrazolo[1,5-a]-pyrimidin-3-yl]phenol
pIGF2: precursor IGF2
PR: Progesterone Receptor
qPCR: quantitative-real-time PCR
TNBC: Triple negative breast cancer
